# Urban economic fitness and complexity from patent data

**DOI:** 10.1038/s41598-023-30649-1

**Published:** 2023-03-04

**Authors:** Matteo Straccamore, Matteo Bruno, Bernardo Monechi, Vittorio Loreto

**Affiliations:** 1 Centro Ricerche Enrico Fermi, Via Panisperna 89/A, 00184 Rome, Italy; 2 Physics Department, Sapienza University of Rome, Piazzale Aldo Moro 2, 00185 Rome, Italy; 3 Sony Computer Science Laboratories Paris, 6, Rue Amyot, 75005 Paris, France; 4 Sony Computer Science Laboratories Rome, Joint Initiative CREF-Sony, Centro Ricerche Enrico Fermi, Via Panisperna 89/A, 00184 Rome, Italy

**Keywords:** Complex networks, Environmental social sciences

## Abstract

Over the years, the growing availability of extensive datasets about registered patents allowed researchers to get a deeper insight into the drivers of technological innovation. In this work, we investigate how patents’ technological contents characterise metropolitan areas’ development and how innovation is related to GDP per capita. Exploiting worldwide data from 1980 to 2014, and through network-based techniques that only use information about patents, we identify coherent distinguished groups of metropolitan areas, either clustered in the same geographical area or similar in terms of their economic features. Moreover, we extend the notion of coherent diversification to patent production and show how it is linked to the economic growth of metropolitan areas. Our findings draw a picture in which technological innovation can play a key role in the economic development of urban areas. We contend that the tools introduced in this paper can be used to further explore the interplay between urban growth and technological innovation.

## Introduction

Modern cities are at the centre of a passionate debate about their future. With over 55% of the global population now living in urban areas, cities represent the core of the modern world. They are important hubs for the production and diffusion of innovation^1,2^, and they play a pivotal role in the diffusion of science^3^ and culture^4^. The ongoing COVID-19 pandemic has placed unprecedented stress on urban infrastructure and has highlighted the need to rethink the role of cities in urban planning and policy decisions. While urbanisation keeps thriving across the world^5^, the challenge of understanding the development of cities to make them more sustainable and resilient becomes more and more crucial^6,7^. Even in urbanised areas that have stabilised their populations, the decarbonisation goal will require cities to adapt and evolve. Therefore, it is paramount to tackle urban areas’ challenges by going beyond pure optimisation schemes and keeping a dynamic perspective. New tools are thus needed to understand and map the present and forecast how a change in the current conditions will affect and modify future scenarios.

Despite belonging to different geographical areas and socio-economic contexts, cities possess general features for economic development and urbanisation rates. For example, many urban socio-economic indicators display power-law correlations with the population size^8^. Hong et al.^9^ observe how US cities with different sizes show a different kind of economic activity, with those with a population of more than 1.2 million capable of sustaining an innovative economy. However, cities are ever-evolving systems where several changes and different growth paths are possible^10^. Technological innovation is often highlighted as one of the main drivers for evolution and change in cities, and it has been shown that complex economic activities flourish in large urban areas^11^, although remote working and dispersed research teams can mitigate the concentration of innovation in urban areas^12–15^. In parallel, many studies recently focused on how innovation diffuses temporally and spatially^16–18^, also with a particular focus on cities^19^. In this paper, we focus on *technological innovation*, and we investigate how the technological fingerprints of cities can affect their development and potential.

For the past few decades, patent data have become a workhorse for the literature on technical change, mainly due to the growing availability of data about patent documents^20^. This ever-increasing data availability (e.g., PATSTAT, REGPAT and Google Patents^21^) has facilitated and prompted researchers worldwide to investigate various questions regarding the patenting activity. For example, the nature of inventions, their network structure and their role in explaining the technological change were broadly investigated^20,22,23^.

It is also important to point out the limitations of using patents as a proxy for innovation^24^. For instance, it has been argued that the disadvantage of using patents is that it is difficult to estimate their value^25^: there are many, if not most, patents with little market value, while some may be of significant value. At the same time, the disadvantages of patent statistics as an aggregate measure of economic and inventive activity are well known^26,27^. It should be clear that inventions do not represent all forms of knowledge production in the economy, nor do patents cover all generated knowledge^28^. Also, patents represent only one of many knowledge indicators and do not capture all sectors in the economy equally^29,30^.

The adoption of patent data to monitor technological innovation is however well established in the literature^27,31,32^. One of the decisive advantages of using patents is the presence of codes associated with the claims contained in the patent applications. These codes mark the boundaries of the commercial exclusion rights demanded by inventors. Claims are classified based on the technological areas they impact according to existing classifications (e.g., the IPC classification^33^) to allow the evaluation by patent offices. Mapping claims to classification codes allows localising patents and patent applications within the technology space. Many studies recently relied on network-based techniques to unfold the complex interplay among patents, technological codes and geographical reference areas. Network science techniques allowed to analyse economic activities of countries^34^, regions^35–39^, cities^2,40–42^ or firms^43,44^.

The purpose of this paper is to measure urban technological innovation using the Economic Fitness and Complexity approach and investigate its correlation with economic growth in cities, as the two have already been found to be related^45^, but we do not aim at providing a holistic description of innovation. On the contrary, the use of little information is the main strength of the Economic Fitness and Complexity method. Using patent data and network techniques we investigate the technological innovation processes happening in cities and, in general, urban areas. Similar approaches were used before for the study of local geographical technological spillovers. However, often the areas considered were regional^35,46,47^, although a similar study, restricted to US cities and with a different metric for complexity, was conducted by Balland and Rigby^48^.

We summarise our research questions as follows:

*Which cities have the most advanced technological production?* We use the framework of Economic Fitness and Complexity (FC)^49^ to quantify the complexity of metropolitan areas and their technological endowment. Introduced initially and extensively employed for countries’ production/exports^49,50^, the approach can be extended to any bipartite system, as in our case, technological production in urban areas. This approach has been adopted by several different international institutions and continues to raise interest in policy-making^51^. Let us note that technological Fitness has already been used in previous studies^48,52^.

*Are cities able to diversify their production of patents, or do they tend to specialise in particular sectors?* In economics, FC has also been applied to sub-national scales, such as regions^53–55^ and firms, both at a country^56^ and global^57^ level. The study of bipartite economic systems at different scales revealed that to apply the FC framework, the economic agents need to have the capability to diversify to create global competition in the system. Otherwise, they will try to specialise and create a nested subsystem of entities specialising in the same products. In such a case, the FC can capture the interplay among the economic agents, provided that the analysis is restricted to subsystems. In this sense, the scale of the system is fundamental and regulates the interplay between competition and specialisation. We aim to understand whether metropolitan areas can compete globally or if they tend to specialise.

*Are there clusters of cities with similar technological baskets?* Starting from a bipartite system linking metropolitan areas and technology codes, we investigate the relations and similarities among metropolitan areas and uncover meaningful patterns in the evolution of their technological production. In bipartite systems, it is often important to understand the similarities between pairs of nodes of the same layer, to obtain a validated projection on a single layer^58^. We adopt this procedure to understand which metropolitan areas are more similar in the type of patents they produce and which patents are more likely to be produced together.

The paper is organised as follows: in “Data”, we describe the data used in this work and we go through our data cleaning procedure. In “Methods”, we introduce the methodologies used in our work, describing the details of the networks and measures we employed. In “Results”, we discuss the results showing how the network techniques can highlight non-trivial clusters of technologies and metropolitan areas, and how both Fitness and coherent diversification are linked to a higher increase in the GDPpc of metropolitan areas. Finally, “Discussion” sums up our contributions and hints at future work needed to address questions arising from this study.

## Data

### Technology codes

Here, we shall adopt the PATSTAT database (www.epo.org/searching-for-patents/business/patstat) that provides information about patents and technology codes. The database contains approximately 100 million patents registered in about 100 Patent Offices. Each patent is associated with a code that uniquely identifies the patent and a certain number of associated technology codes. The WIPO (World International Patent Office) uses the IPC (International Patent Classification) standard^33^ to assign technology codes to each patent. IPC codes make a hierarchical classification based on six levels called digits, progressively providing more details about the technology used. The first digit represents the macro category: for example, the code Cxxxxx corresponds to the macro category “Chemistry; Metallurgy” and Hxxxxx to the macro category “Electricity”; considering the subsequent digits, we have, for instance, with C01xxx, the class “Inorganic Chemistry” and with C07xxx the class “Organic Chemistry”. We assign a year to each patent based on the first filing date.

After assigning a technology code and year to each patent, we use a database about cities (see next section) to match the unique patent identifier and its technology code to the corresponding city. To geolocalise the patents, we adopt the De Rassenfosse et al. database^59^ that contains entries on 18 million patents from 1980 to 2014. In this database, the geographical information of patents is conveniently assigned to precise geographical coordinates. Thus, each patent has a unique identifier, a series of technology codes, and geographical coordinates identifying the corresponding city. In the Supplementary Information, we describe the importance of the De Rassenfosse et al. work, and we summarise some useful features of this database.

### GDP of cities

To obtain information on the GDP of cities and their evolution, we used the work of Kummu et al.^60^. The authors constructed a worldwide GDP grid with a resolution of about five arc minutes for the 25 years 1990-2015. To compute the GDP per capita of each city or metropolitan area (MA) for each year in the data, we first download the boundaries from the Global Human Settlement Layer^61^. Considering the GDP grid in one year, we compute the GPD per capita of a MA as the average of all the grid points within its boundaries. In Fig. 3 in the Supplementary Information, we show the example of the grid of the Rome metropolitan area. In Supplementary Information also, we quantify the relative error due to the grid calculation, considering as a proxy the OECD GDPpc data.

### Data cleaning procedure

To clean the data, the first step is to associate the technology codes of a patent with a specific city. Once this preliminary operation is completed, it is possible to build the bipartite networks that will link cities to technology codes. We represent the bipartite networks through bi-adjacency rectangular matrices $V^y$ whose elements $V_{c,t}^y$ are integers indicating how many times a technology code *t* appeared in different patents in a given city *c* in the year *y*. In total, our network features 42912 cities connected to 650 technology codes (4-digit). To reduce the difference between the two layers of the networks and reduce the noise in the system which is often due to the presence of very small cities, we aggregate the cities in the respective metropolitan areas (MAs). We select all cities within a metropolitan area (MA), and the technology codes associated with the metropolitan area will be the union of all the technology codes of the cities within it. The MAs present in the Global Human Settlement Layer^61^ are 8641 and cover the entire world. However, most of these do not contain cities that have patents. The metropolitan areas producing patents are 2169 and are distributed as shown in Figs. 4 and 5 in the Supplementary Information.

We obtain a matrix ${\textbf {V}}^y$ for each year *y* from 1980 to 2014, connecting 2169 metropolitan areas *a* and 650 technology codes *t*. To avoid the fluctuations due to using only one year at a time as an interval, we decided to consider a window of 5 years each time, summing the matrices in one window. In this paper, therefore, the matrix ${\textbf {V}}^y$ will refer to the time window from *y* to $y+5$. The final database consists of 30 5-year window matrices ${\textbf {V}}^y$ ranging from window $1980-1984$ to 2010–2014. Finally, we binarise the matrices *V* applying a standard procedure in economic complexity to determine relevant producers/exporters of products (see “Revealed comparative advantage”).

**Figure 1:**
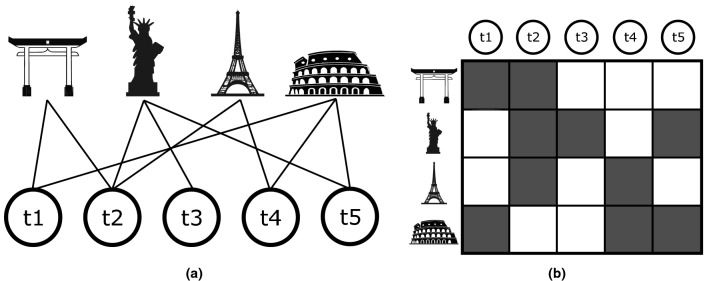
Bipartite metropolitan areas—technology codes network. **(a)** Pictorial representation of the bipartite metropolitan areas-technology codes network. Each MA is connected to one or more technology sectors. **(b)** Pictorial representation of the bipartite network adjacency matrix. A dark square means that a given technology code is present in a patent made by a given MA, and therefore a link is present in the bipartite network.

## Methods

### Revealed comparative advantage

To understand which metropolitan areas are relevant innovators of a specific technological sector, we apply the revealed comparative advantage (RCA)^62^ binarisation strategy. RCA is a frequently used tool in the economic complexity literature^34,50,63^. Considering a bipartite network of countries and products, RCA allows us to determine how competitive a country is in exporting a given product while also considering how many countries export that product. In our case, RCA reveals when the share of patents of some technology, *t*, introduced by a certain MA, *a*, is higher than the average share of the rest of the market, meaning that the metropolitan area focuses on the technology *t* more than the number of technologies produced would suggest. The RCA metric is similar to the Location Quotient^64^, which is used to measure the concentration of a certain industry in a particular region^65^.

Considering the matrix ${\textbf {V}}^y$ for the year *y*, we define the RCA for the MA *a* and the technology *t* as:

$$\begin{aligned} {RCA^y_{a,t}} = \frac{{V_{a,t}^y}/\sum _{t'}{{V_{a,t'}^y}}}{\sum _{a'}{{V_{a',t}^y}}/\sum _{a',t'}{{V_{a',t'}^y}}}, \end{aligned}$$

A value $RCA_{a,t} \ge 1$ means that MA *a* is significantly competitive in the technology field *t*. We use this threshold on the RCA values to obtain 30 ${\textbf {M}}^y$ matrices, one for each 5-year window:

$$\begin{aligned} M^y_{a,t} = {\left\{ \begin{array}{ll} 1\ \text {if}\ RCA^y_{a,t} \ge 1\\ 0\ \text {if}\ RCA^y_{a,t} < 1. \end{array}\right. } \end{aligned}$$

Notice that, in the following, we consider only having an average of at least one RCA $\ge 1$ per year, reducing their number to 1211. These ${\textbf {M}}^y$ matrices represent our final temporal bipartite network that links 1211 MAs to 650 technology codes.

### Bipartite networks

A bipartite network is a network whose nodes represent two different kinds of entities, and only connections between nodes from different entities are allowed. Many systems in ecological and socio-economical environments, such as those studied in the present work, are easily described as bipartite since they involve interactions between two kinds of entities^56,66^. For instance, the Internet can be modeled as a users-websites bipartite network, whose analysis can reveal sets and ranks of pages that will be more likely to be of interest for the user^67^. We use the ${\textbf {M}}^y$ matrices as bi-adjacency matrices of MA - technology bipartite networks, connecting each MA with the technologies in which it is competitive. In Fig. 1 we show a pictorial representation of this bipartite network and its bi-adjacency matrix ${\textbf {M}}^y$ for the year $y = 2000$.

In bipartite systems, it is often important to study which nodes belonging to the same layer are similar. In our case, this means finding MAs whose basket of patents is similar or technologies that are developed in a common set of MAs. In order to find the similarities among MAs and technologies, we project the bipartite network onto its layers, obtaining two monopartite similarity networks of MAs and technologies. However, the problem of finding the proper projection of a bipartite network into a monopartite one representing the non-trivial similarities of nodes belonging to one of its layers is well-known in the literature^58,67–70^. In general, the goal is to find a monopartite network that best represents the bipartite one without taking too much information away from the latter. We decided to use the bipartite configuration model (BiCM)^71,72^ to select the most significant links in the projected networks.

#### Bipartite configuration model (BiCM)

One of the simplest ways to obtain a monopartite projection from bipartite data is to count the number of links in common between two different entities belonging to the same layer. However, in the case of non-sparse real networks, this procedure often yields a densely connected projection with a trivial topology, not highlighting relevant patterns.

Instead, we need to keep only links representing significant similarities between nodes in our projected networks, to avoid obtaining a too dense projection; many algorithms have been proposed for filtering dense projections^58,70,73^. We follow the procedure described in^68^, using as a null model the Bipartite Configuration Model (BiCM)^71^, which we compute by using the *NEMtropy* Python package (github.com/nicoloval/NEMtropy)^72^. Simply put, the BiCM is a null model for bipartite networks that can be used as a tool to compare the observed network with the one that would be expected from the degree sequences of the nodes, i.e. the number of connections of each agent in the system. Belonging to the family of maximum entropy random graphs, BiCM yields a maximally unbiased probability distribution over an ensemble of networks with the same number of nodes of the observed network. The model outputs a link probability $p_{i\alpha }$ for each pair of nodes *i*, $\alpha$ belonging to opposite layers. Then, the link probabilities can be used to sample from this ensemble, simply treating each link as a Bernoulli random variable with parameter $p_{i\alpha }$, or to compute the expected quantities in the network. In this way, the properties of the observed network can be compared with the benchmark provided by the BiCM, to understand if the former is statistically explained by the number of connections of the agents of the system.

#### Network projection

A practical application is the use of BiCM as a filter for projections of a bipartite network on one of its layers. We compare the observed number of shared connections $V_{ij}^* = \sum _\alpha M_{i\alpha }M_{j\alpha }$ for each pair of nodes *i*, *j* belonging to the same layer, to the same quantity expected by the null model. In our case, we compute the p-value of $V_{ij}^*$ with respect to the probability distribution of the model. Then, we apply a statistical test to keep only the links between the pair of nodes whose respective p-value results are smaller than a statistically significant threshold. We use the False Discovery Rate (FDR) test, which is commonly used in the case of repeated hypotheses testing^74^. The threshold of the FDR represents the statistical significance of the whole projected network. This projection procedure is similar to what is also called Stochastic Degree Sequence Model^75^.

Applying the described method, we obtain a monopartite projection of the original bipartite network that is statistically significant, i.e. keeps only the relevant similarities with respect to the degrees of the nodes, unveiling hidden patterns. We do this to obtain networks of similarities for both MAs and technologies. In the Supplementary Information, we describe the technical details of the methodology for the computation of the BiCM and the monopartite projection.

We apply the projection procedure for each bipartite network of MAs and technologies built for each 5-year time window. Then we aggregate the monopartite projections obtained for each year into a single cumulative one, that captures the similarities among MAs and among technologies. We perform this aggregation by summing all the network projection adjacency matrices for each time window. For instance, suppose node *i* is connected with *j* in the projection relative to 1980–1984, but node *k* does not appear in this network; suppose also that in the network of 1990–1994, *i* is connected with *k*, but *j* does not appear. In the merged network, we will have both a link between *i* and *j* and a link between *i* and *k*. We keep multiple links as weights: e.g., if two nodes are connected in three projected networks corresponding to three different time windows, the relative link weight will be 3, emphasising relevant similarities that last over time.

#### Community detection

We are interested in finding relevant communities of MAs or technologies to visualise better which nodes in the two layers are highly interconnected. Broadly speaking, a community is a subset of nodes in a network that is more densely connected than expected: for example, a group of close friends in a social network can be a community. To find communities in the networks of similarities obtained via the projection of the original network, we adopted the Louvain method introduced by Blondel et al.^76^, which relies on finding a partition that maximises the modularity, a very well-known quantity in complex networks. Put simply, modularity is a global measure of a partition of nodes in a network that captures how much the partition is able to describe the communities in the network. Thus, maximising modularity means finding the partition that captures the community structure of the network.

We also vary the resolution parameter^77^ in the modularity optimisation, which can be exploited to find communities at different scales.

### Fitness and complexity algorithm

The fitness and complexity (FC) framework^49^, introduced in 2012, provides a way to quantify the competitiveness (Fitness) of the economy of a country. Here, we adopt it to quantify the Fitness of metropolitan areas considering only patent data. The idea is to define an iterative process linking and combining the Fitness of a MA, $F_a$, with the Complexity of a specific technology, $Q_t$. The iterations to find these quantities are defined as:

1 $$\begin{aligned} {\left\{ \begin{array}{ll} \tilde{F}_a^{n+1} = \sum _t{M_{at}Q^{n}_t}\\ \tilde{Q}_t^{n+1} = \frac{1}{\sum _a{\frac{M_{at}}{F^{n}_a}}} \end{array}\right. } \end{aligned}$$

where for each step *n* the quantities are normalised as:

2 $$\begin{aligned} {\left\{ \begin{array}{ll} F^n_a = \frac{\tilde{F}_a^n}{\left<\tilde{F}\right>_a}\\ Q_t^n = \frac{\tilde{Q}_t^n}{\left<\tilde{Q}\right>_t} \end{array}\right. } \end{aligned}$$

and initial conditions $Q_t^{(0)} = 1\ \forall t$, $F_a^{(0)} = 1\ \forall a$. The convergence of the algorithm has been studied extensively^78^. In our case, we compute $F^y_a$ and $C^y_t$ for each 5-year window *y* starting from the bi-adjacency matrices $M^y_{at}$. We stop the iteration when the Fitness ranking of MAs does not change anymore. The rationale behind the whole process is as follows. A technology made in an already developed MA carries little information about the complexity of the technology itself because developed metropolitan areas produce a large part of the technologies. In contrast, a technology exported by an underdeveloped MA must require a low level of sophistication. Thus, it is possible to measure a MA’s technological competitiveness given the complexity of its technologies. Instead, a different approach should be taken to assess product quality. Fitness $F_a$ is proportional to the sum of technologies, weighted by their complexity $Q_t$. Intuitively, the complexity of a technology is inversely proportional to the number of MAs that have implemented it. If a MA has high Fitness, this should reduce the burden of limiting the complexity of a technology, and MAs with low Fitness should contribute strongly to $Q_t$.

Recent studies have shown that it is helpful to calculate the Fitness of sub-national actors using the complexity that comes from the national systems^54,79,80^. This measure is called exogenous Fitness and overcomes the issue of the limited capabilities of sub-national entities, such as cities or MAs in our case. This exogenous metric acts as an instrumental variable, to keep the results more consistent over the years and compare different sub-national entities, whose scale can be very different. Thus, for Fitness calculations, we use the complexity obtained by considering global international patent data instead of calculating the complexity of a technology only on the MA subsample. We proceed in the same way by aggregating all the MAs of a country, i.e., summing all the rows of the MAs and running the FC algorithm. In other words, we compute $F^C$ and $Q^C$ relative to each country *c* and technology *t* through the formulas 1, and then calculate the Fitness of the MAs through:

$$\begin{aligned} F^{MA}_a = \sum _t{M_{at}Q^C_t}. \end{aligned}$$

For each time window, we calculate the Exogenous Fitness of all metropolitan areas and the complexity of each technology.

### Coherent diversification

**Figure 2:**
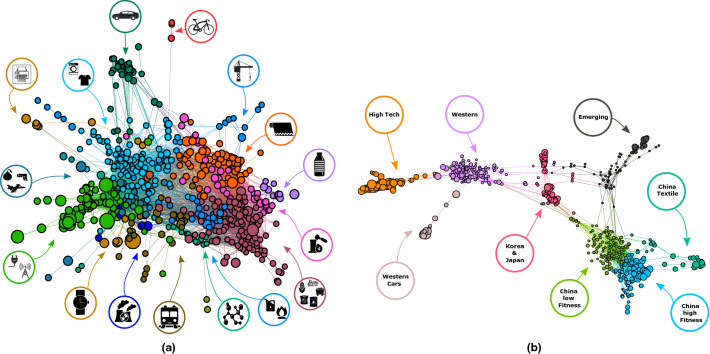
Monopartite projections of the metropolitan areas-technology bipartite network. **(a)** Technology network. Each node in this figure is a technology code. The size of the nodes is proportional to the complexity of the technology. We could identify the specific significant technologies for most clusters and represent them with corresponding icons in coloured circles. The Electricity and Information cluster (light green on the left) contains the most complex technologies. **(b)** Metropolitan areas network. Each node in this graph is a MA and each node size is proportional to the respective fitness. The high-tech cluster is the one containing the MAs with the highest fitnesses.

The coherence of production and innovation diversification has been shown to be a significant driver of productivity^81,82^. Thus, to better understand the nature of MAs’ performance from their technology portfolio, we analyse their coherent diversification^44^. The underlying question is whether the accumulation of knowledge and capabilities associated with a coherent set of technologies leads MAs to experience more significant benefits in terms of GDPpc. Consistent diversification is defined as the Coherence of the technology field *t* with respect to the technology basket of the MA *a*:

3 $$\begin{aligned} \gamma _{at}=\sum _{t' \ne t}{B_{tt'}M_{at'}}. \end{aligned}$$

where *B* can be any matrix quantifying the similarities between pairs of technologies and *M* is the usual adjacency matrix of a bipartite network between the layers of MAs and technologies. For each technological field, *t*, and each MA, *a*, one counts how many technologies $t^{\prime }$ adopted by *a* are connected with *t*, using $B_{tt^{\prime }}$ as a weight. If the technological portfolio of *a* is such that *t* is surrounded by numerous strongly connected technologies owned by *a*, then *t* will be very coherent to *a*, and $\gamma _{at}$ will be high. On the contrary, if *t* belongs to a portion of the network of technologies far from the patenting activity of *a*, $\gamma _{at}$ will be low. In our case, we use as *B* matrix the projection represented in Fig. 2a. Notice that $\gamma$ has the same dimensions as *M*, and the elements quantify how coherent a technology *t* is to the technology basket of MA *a*.

Finally, we can calculate the coherent technological diversification^44^ of MA, *a*, as:

4 $$\begin{aligned} \Gamma _a = \frac{\sum _{t}{M_{at}{\gamma _{at}}}}{d_a}, \end{aligned}$$

where $d_a = \sum _t{M_{at}}$ is the diversification of MA, *a*. The Coherence of technological diversification, $\Gamma _a$, of MA *a* computes the average Coherence $\gamma$ of the technologies in which *a* is patenting.

## Results

### Networks of similarities of MAs and technologies

To find a general network representation of our data for each year, we project each bipartite network of MAs and technologies, one for each 5-year window (for both layers of technology codes and MAs) in our dataset. Then we aggregate all the monopartite projections of each year to obtain a cumulative description of the similarities among the nodes. On these final similarity networks, we find groups of similar products by performing community detection. The detailed steps for obtaining the projections and finding the communities are described in the Methods section and in the SI. The resulting networks are shown in Fig 2.

The technology network of Fig. 2a does not show a strong modular structure due to the ability of MAs to produce patents in different areas, yielding instead different communities but with contiguous clusters containing products of similar macro-type. For instance, we can find the technology communities of (clockwise, starting from the left/light green) communication & information, weapons, printers, domestic technologies, cars, bicycles, buildings, textile, plastic, metallurgy, agri-food & mining, fuels, organic chemistry, trains, nuclear energy, and clocks-related technologies. Node sizes are proportional to their complexity. The cluster with the highest number of complex nodes is the communication & information one, pointing out that not all MAs have the necessary capabilities to patent in this area.

The statistically validated projection representing the similarity network of metropolitan areas shows how MAs can be related from the point of view of technological production, and we can see how both the location and the development of cities can be a factor to their similarity. We find well-defined communities of MAs that we can label as Chinese, emerging countries, Euro + US, Japanese + Korean MAs, car-related, high-tech. The high Fitness cluster related to high-tech production contains MAs such as London, New York or San Jose, while the community made by Western car manufacturing MAs includes for instance Turin, Detroit, Stuttgart. Interestingly, the Japanese & Korean cluster shares connections with both the western, the Chinese and the emerging countries clusters. This last one contains MAs from Mexico, Brazil, India, Hungary, and also some metropolitan areas from rich countries but with a growing economy, often related to manufacturing. The Chinese clusters are three, specifically one is more devoted to textile production, while the other two are close, but one contains generally higher Fitness metropolitan areas. We present a complete table of MAs with their class in the Supplementary Information.

### Fitness-GDP relation in metropolitan areas

**Figure 3:**
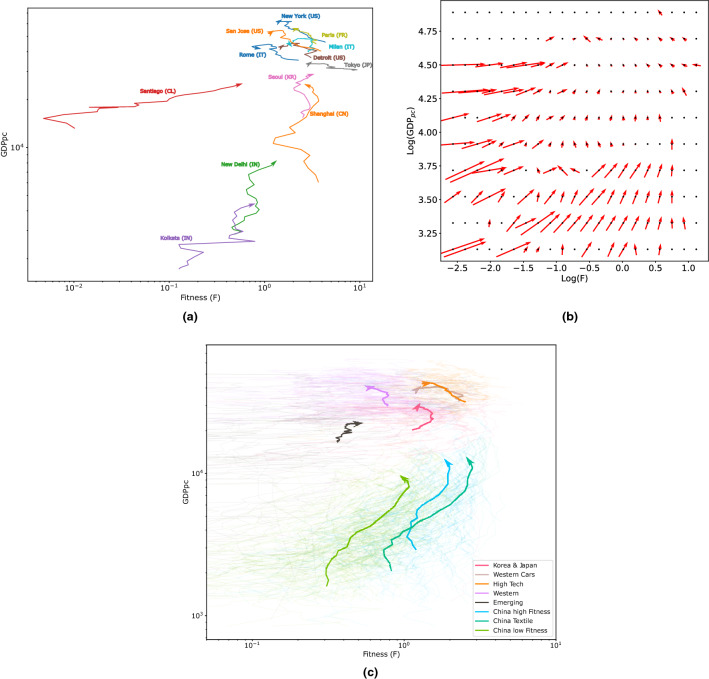
The Fitness-GDPpc plane in the case of metropolitan areas and their technological production. **(a)** We trace the trajectory of some MAs from 1990 to 2010 in the Fitness-GDPpc plane. MAs with high Fitness show a more significant increase in GDPpc. (**b**) We show an average vector field of the trajectories from 1995 to 2005. In this plot, we can better visualise how high Fitness is correlated to increases in GDPpc, most notably in the lower right part of the plot. In contrast, MAs with low Fitness will tend to increase it first. **(c)** Trends of all MAs, with trajectories coloured according to the community of belonging. For each community, we highlighted the average trajectory.

In Fig. 3, we report the results regarding the relationship between the technology basket of MAs and their GDPpc.

We apply the Fitness and Complexity algorithm described in the Methods section: we first calculate the complexity of technologies at the country level and then compute the exogenous Fitness of the MAs. In Fig. 3 we report three different representations of the GDPpc-Fitness plane. In the first (a), we trace the trajectories of some MAs from 1990 to 2010. Metropolitan areas with high Fitness are generally more likely to have a more significant increase in GDPpc. For Shanghai, for instance, the trajectory is nearly vertical, ending at a similar value of GDPpc as Santiago. Santiago is also an interesting case as its trajectory moves in an almost horizontal line increasing the Fitness but cannot improve the GDPpc quite as much as Shanghai. Other MAs, such as the Indian New Delhi and Kolkata, also tend to grow consistently in Fitness and GDPpc. The same phenomenology is mirrored in Fig. 3 panel b, where we show the average vector field of the trajectories from 1995 to 2005. From this plot, it emerges that metropolitan areas with a high Fitness generally show an increase in GDPpc, except for those that already have a very high GDPpc. Finally, in Fig. 3, panel c, we show the overall trend of all MAs whose trajectories are coloured according to the community of belonging. For each community, we highlight the average trajectories. The three communities of Chinese MAs are particularly interesting since they show similar trends of Fitness increase. The other clusters show different trends that can be easily interpreted in terms of GDPpc and Fitness. The *High-Tech* cluster has the highest average GDPpc, while the *Western* and *Western cars* ones have the same average GDPpc with the difference that the latter has higher Fitness. The Korea & Japan cluster has a low average GDPpc compared to the previous three clusters, though with a comparable Fitness. The cluster labeled as Emerging is slowly increasing in terms of average GDPpc and Fitness, but its increase in GDPpc seems to be halted, differently from the Korea/Japan one. The Fitness trends of all clusters are decreasing, except for the Emerging and Chinese clusters. This behaviour is justified by considering that Fitness is a globally computed quantity, using data about all MAs. For this reason, the Fitness cannot increase for all MAs simultaneously, and if it increases for some MAs, it must automatically decrease for others.

Let us note that, as highlighted by Balland et al.^51^, our approach is a “phenotypic” one, meaning our analysis and results on Fitness were obtained by using only information about metropolitan areas and patents. This, in general, can be a non trivial limitation because we do not perform a “genotypic” analysis, i.e. we don’t consider the knowledge of the capabilities to make the technologies and the processes by which MAs can use their abilities to produce them. This would go beyond the scope of the present work and would indeed require much more data; although this can be seen as a limitation, one of the most crucial points for our measure is exactly that it needs little data to be computed.

### Fitness innovation rankings of metropolitan areas

**Figure 4:**
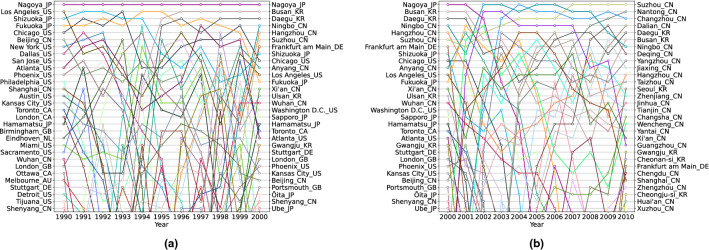
The Fitness rankings of metropolitan areas. The 30 MAs with the highest Fitness are shown, along with the evolution from 1990 to 2000 **(a)** and from 2000 to 2010 **(b)**. In 1990 many of the metropolitan areas in the top 30 of the Fitness rankings were from the US, Europe, Canada and Japan, with only Shanghai and Beijing from China in the first 20. In 2000, Chinese and Korean MAs appear in the top 30, and in 2010 they dominate the top of the Fitness rankings with Frankfurt as the only European, 6 Korean MAs and all others being Chinese.

The metropolitan areas with the highest Fitness per year are presented in Fig. 4. It is remarkable, even in this case, the rise of Chinese MAs from 1990 to 2010: at first, only the biggest areas such as Beijing and Shanghai enter in the top 30 of the Fitness rankings. Nagoya (Japan) sits atop of the rankings from 1990 to 2001, then it is overtaken by the wave of Chinese cities that start to monopolise the top 30 shortly after 2000; in 2000 the rankings are still mixed, including many Chinese metropolitan areas but also still many from the US and Japan. Ten years later, there are only seven metropolitan areas in the top 30 that are not Chinese: six of these are Korean and only one is European, Frankfurt. In 2020 Suzhou tops the rankings, followed by other Chinese metropolises such as Nantong, and the first non-Chinese MAs are the Korean Daegu and Busan, which were also at the top in the 2000 rankings.

This impressive surge of the patents made by Chinese metropolitan areas appears to have been strategically coordinated and aimed at rapid development and modernisation of the country, even if recent studies have deeply analysed and in some measure criticised the increase in China’s patenting activities^83–85^.

### Coherent technology production

**Figure 5:**
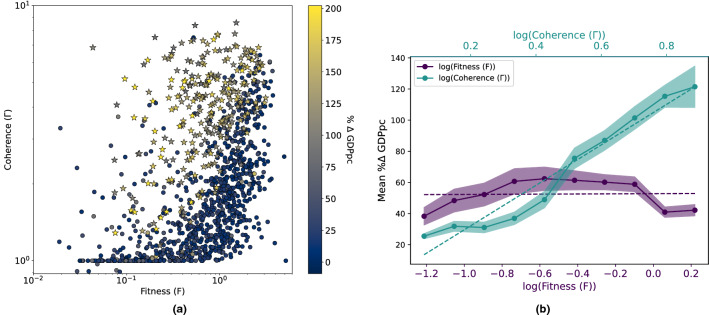
Fitness VS coherence to evaluate GDPpc growth. **(a)** Fitness–coherence plane. We represent the averages of the measures over the decade 1995–2005, and the colour scale is the fractional change of GDPpc over the years. We observe how the Coherence allows discriminating MAs with a more significant positive change in GDPpc. Stars indicate the Chinese MAs. **(b)** Average fractional change of the GDPpc versus Fitness and Coherence. To highlight that Coherence correlates better with changes in GDPpc, we divide Fitness and Coherence into ten bins and calculate the mean fractional GDPpc variation of all the points in each of the ten bins. The Fitness curve is roughly constant, highlighting that Fitness cannot discriminate different fractional changes of the GDPpc; this is in part due to rich metropolitan areas with high Fitness and with a rather stable GDPpc. Coherence, instead, displays a growing trend with the fractional change of GDPpc, i.e. the higher the Coherence, the higher % $\Delta$ GDPpc.

In Fig. 5 we show the results of coherent diversification in technological production. From Fig. 5 panel a, displaying the Coherence–Fitness plane, we observe that Coherence correlates with a positive change in the GDPpc of MAs better than Fitness. Fig. 5 panel b confirms this picture: while the change in GDPpc is not sensitive to Fitness changes, a growing trend of Coherence is accompanied by a parallel growth in the GDPpc’s change. High Fitness MAs are generally rich and with a stable GDPpc. This result is appealing, especially if we consider that, in the Coherence rankings, 79 MAs out of the top 100 are Chinese. The coherent diversification strategy of China was already highlighted in a previous work by Gao et al.^86^, who noticed similar coherent patterns for the expansion of the production in Chinese regions. To ensure that our result is not simply due to the relatively high number of Chinese MAs in our dataset, we performed a robustness test, described in more detail in the Supplementary Information. In this test, we rebuild the technology network as explained in the “Networks projection” Section without the Chinese MAs, to then compute the Coherence using all MAs. In the Supplementary Information, we also ran a simple check to show that a high Coherence is not related to low diversification. In the Supplementary Information, the interested reader can find a histogram to show the mean Coherence of the different MA clusters.

## Discussion

In this work, we studied technological innovation in metropolitan areas by analysing data on patent production. In particular, we focused on the signals of specialisation and diversification by applying the Fitness and Complexity framework and novel methods for bipartite networks to the technological production of metropolitan areas. The Fitness and Complexity algorithm is particularly suitable in this context because the interplay between specialisation and diversification can change at different scales^57^.

Our findings indicate that MAs tend to specialise in technology sectors, particularly for some technological categories, such as cars or electronics, but the biggest ones are able to diversify and some manage to be more generalists, although their focus shifts to complex technologies. Moreover, we observed similarities among metropolitan areas within a country or across similar countries. Chinese MAs give the best example of similar MAs in a single country. They are organised in three coherent clusters specialised in similar technological baskets. The coherent diversification strategies of China are in line with previous results analysing technology spillovers in Chinese regions^86^. One of the clusters is specialising in the technology sectors of textile industries, another one specialises in agri-food and the third cluster is devoted to highly sophisticated technology sectors. We observe a similar behaviour of relatedness, though at a smaller scale, in Japanese and South-Korean MAs. Our work also highlighted similarities among emerging MAs and among highly technological metropolitan areas. Interestingly, the network of similarities among MAs shows a clear geographical boundary between highly developed Asian and Western (European/American) MAs.

We applied the Fitness and Complexity framework to understand the evolution of the quality in technological innovation of MAs and their clusters. In line with previous results, we have shown that a high Fitness can be correlated with a high GDP per capita: MAs with a complex technological basket show higher increases in GDPpc in the following years than MAs developing more basic technologies. Korea and Japan followed this path, especially in past years. In recent years, the standout case is China: the complexity of innovation in Chinese MAs is very high, and their GDPpc displays rapid growth. We found that Chinese metropolitan areas are not only able to diversify their innovation patterns by aiming for a more complex technological basket, but also do this in a coherent and coordinated way. Measuring, in fact, the Coherence of the innovation baskets of MAs, we show that a vast majority of MAs with the highest Coherence values are Chinese, and we report that this outcome is not due to a restriction to a specific set of technologies. On the contrary, Chinese MAs diversify consistently and coherently. Moreover, a coordinated effort is also evident, with Chinese MAs areas sharing common sets of technologies. Our results indicate that coherent diversification is necessary and arguably more decisive than Fitness to increase the wealth of a metropolitan area, as the highest increase in GDPpc is found in metropolitan areas with high Coherence. Diversifying coherently appears to be the most logical path for developing metropolitan areas that need to expand their capabilities.

We found that from 1990 to 2010, the ranking of the top 30 MAs in patent production’s Fitness drastically changed. In 1990, many metropolitan areas from many rich countries were sitting at the top of the table, with Japan and the US vastly represented, Nagoya and Los Angeles in the top two positions, and only Beijing and Shanghai as Chinese metropolitan areas. By contrast, in 2010, only seven metropolitan areas in the Fitness top 30 rankings were not Chinese: six Korean ones and a European, Frankfurt, with the Chinese Suzhou topping the table.

In conclusion, our work gives new tools for analysing the patent production of metropolitan areas and cities. Our contribution can be summarised as follows. The application of the Fitness and Complexity framework to patent production provides a quantitative and a qualitative point of view on technological innovation at the scale of metropolitan areas. Since this methodology only considers patent production data, without making assumptions about external indicators, it is easy to adopt. Rankings are stable and capture both the diversification and the focus on more complex products. Among the most complex patents, we find technologies such as communication and information and nuclear energy, while among the least complex, we find mainly manufacturing technology, chemical products, textiles, and plastic.

Among metropolitan areas, we found many groups of similar geographical areas or similar specialisation patterns, with a geographical gradient going from Chinese MAs to western countries’ MAs and a set of emerging MAs. The most prolific patenting cities in 2010 were almost all Chinese and partly Korean, although the situation was very different 20 years before, with Japan, US and Europe being the most represented. The diversification strategies of China were proven to be very effective, and other countries, such as India, could be on their way to imitating them. In contrast, it might be harder for other MAs in countries with emerging economies to become competitive in patent production as their Fitness level is quite different.

The best strategy for metropolitan areas is to diversify coherently with their current technological knowledge. The growth in patent production in Chinese cities gives the best example of this phenomenon. Metropolitan areas with similar patent production baskets are either close geographically or focus on specific technologies in which they can diversify. For example, high-tech technologies can be produced only by a few MAs with a large set of capabilities, thus a high Fitness. Cities that produce car-related technologies are usually similar to each other as they are required to have a particular set of capabilities and they have high Fitness.

### Future work

The theoretical framework presented here can be applied in several scenarios we detail below.

#### Optimal diversification strategies and technology forecasting for MAs at different scales and capabilities

Our methods can be applied to study the best diversification strategy for MAs, assessing the best technologies to develop in a city, as done in previous works^43,87^. However, the ability of a metropolitan area to diversify its technology products depends on its size and capabilities. Large MAs with resources comparable to a whole country can diversify as much as they wish. At the same time, smaller MAs may have different levels of resources and may be limited in their ability to diversify. *Specialisation* and *diversification* are both feasible ways for MAs to compete, depending on their resources and on whether they act more like a large firm^57^ or a whole country^49^. Our results add evidence to the importance of coherent diversification.

#### The strategy of Chinese MAs

We found that the Chinese MAs have the most *coherent* technology diversification and specialisation strategies. These results align with previous work^88^, but the cause of the observed structured diversification remains unanswered: is this behaviour coordinated at the national level? Even though patent production does not capture all the details of China’s growth^83–85^, a more detailed analysis of the Chinese case could highlight whether China is implementing a long-term, all-purpose strategy for developing technologies. For instance, whether China is implementing an a priori definition of production basket for individual MAs. If this is true, can the strategy be copied by other countries, and under which conditions? For instance, some emerging MAs, such as Indian ones, are on a trajectory similar to Chinese MAs, as shown in Fig. 3a for New Delhi.

#### The restricted business of car technologies

The strong signal from MAs dedicated to producing cars is unique and suggests that these metropolitan areas could have trouble diversifying their production. It is not clear yet whether this is a signal of high competitiveness of these kinds of technologies, and therefore MAs should specialise to better profit from this production, or it is hard to implement other technologies for car-focused MAs. However, with the advent of electric cars and considering the significant technological changes about to occur in the forthcoming years (see, for instance, the European ban on fossil-fuel car production by the EU (https://www.euronews.com/green/2022/05/12/eu-wide-ban-on-new-fossil-fuel-cars-to-kick-in-from-2035-as-lawmakers-back-proposal), the future economy of MAs currently producing cars will have to be reshaped. Future studies focusing on optimal diversification strategies and forecasting future technology production could be used to shape technology paths that can help these MAs adapt to such changes.

## Supplementary Information

Supplementary Information.

## Data Availability

A repository of processed data and codes used can be found at: https://github.com/MatteoStraccamore/Urban-Economic-Fitness-and-Complexity-from-Patent-Data. The datasets used in this work for patents and metropolitan areas’ limits are publicly available. The database for patents can be found in PATSTAT (https://data.epo.org/expert-services/index.html). The geolocalisation of patents was provided by the work of De Rassenfosse et al.^59^ (https://doi.org/10.7910/DVN/OTTBDX). Metropolitan areas’ limits are provided by Global Human Settlement Layer (https://data.jrc.ec.europa.eu/dataset/347f0337-f2da-4592-87b3-e25975ec2c95). GDP per capita was extracted from the work of Kummu et al.^60^ (https://doi.org/10.5061/dryad.dk1j0).
