# Supplementary material for: Urban economic fitness and complexity from patent data
Source: Sci Rep. 2023 Mar 4;13:3655. doi: 10.1038/s41598-023-30649-1 (PMC9984762; doi:10.1038/s41598-023-30649-1)
Supplement: Supplementary file 1 — Supplementary Information. [file 41598_2023_30649_MOESM1_ESM.pdf]

Supplementary Information

Urban Economic Fitness and Complexity from Patent Data

Matteo Straccamore, Matteo Bruno, Bernardo Monechi
and Vittorio Loreto

Contents

1 Patents information 3

2 GDP per capita calculation 4

3 MA data cleaning and statistical data visualization. 4

4 Bipartite Configuration Model (BiCM) 5

5 Networks projections 8

6 Metropolitan areas community class table 11

7 Coherence robustness test 13

8 Coherence of clusters 15

1 Patents information

With the database of De Rassenfosse et al. [1], it is possible to geolocate the location of applicants for about 18.9M patents. The goal of this work is to create a dataset of early patent applications from around the world and organise it according to the location of applicants. This allows us to see where the patented inventions originated and where the centres of innovation are located. This information is useful for studying the geography of innovation and for understanding the spatial distribution of patented inventions. It can also be used by policymakers interested in the location decisions of firms and high-skilled workers. Authors perform the geolocalisation by linking the postal codes of applicant addresses to latitude and longitude and, as a result, to countries, regions and cities/MAs. They obtain the postal codes information by matching the addresses present in the patent applications in PATSTAT and other databases that, starting from these address codes, provide the postal codes. We summarise some useful features of this database:

- These patents represent the first application of the invention;
- Some patents are geolocated in multiple locations due to the collaboration of multiple applicants. However, it should be specified that at the end of the assignment of the patents to the respective MAs, about 2% of the total have double or more geolocations;

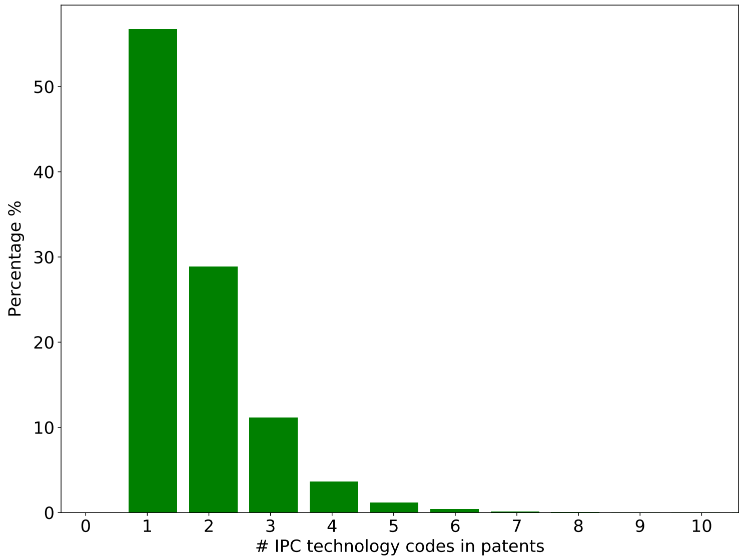

Figure 1: **Percentage of patents in our database VS the number of IPC 4-digits technology codes.** In this figure, we show how more than 50% of the patents available to us are associated with a single technology code. Compressing into 4 digits, the categories become very restrictive.

- Each patent is associated with one or more IPC 4-digit technology codes, and we assign these multiple technology codes, retaining all of them, to patents that have more than one. The categories are already very restrictive and this does not add much noise to the data. In Fig. 1, we show a bar graph showing the percentage of patents in our database that have N IPC 4-digit codes. More than 50% of these have only one technology code.
- Since the database contains information about the first application when multiple patent offices have the same patent, the applicant's position is always the same;
- All patents in the De Rassenfosse database are geolocalised using information from PATSTAT, WIPO, REGPAT, and the Japanese, Chinese, German, French, and British patent offices.

2 GDP per capita calculation

In the work of Kummu et al. [2], authors use data from national and sub-national level to extrapolate and interpolate, respectively if they lack data for the starting/ending years or in the middle years of the database, GDPpc information for missing areas. These operations, however, suffer from some limitations. For example, the interpolation is done with the hypothesis of a smooth trend. In the work, they also reported the error of extrapolation and interpolation methods. To check how good the values of GDPpc obtained by the use of the grid of Kummu et al. ($GDPpc^K$), we report in Figure 2 the relative error obtained considering the GDPpc measure of OECD[1] ($GDPpc^{OECD}$), calculated as $\frac{GDPpc^K - GDPpc^{OECD}}{GDPpc^{OECD}} \times 100$. We do not take the absolute value in the plot to show when the estimate of Kummu et al. is lower than the OECD value. In Fig. 2 we note how indeed there is a good agreement

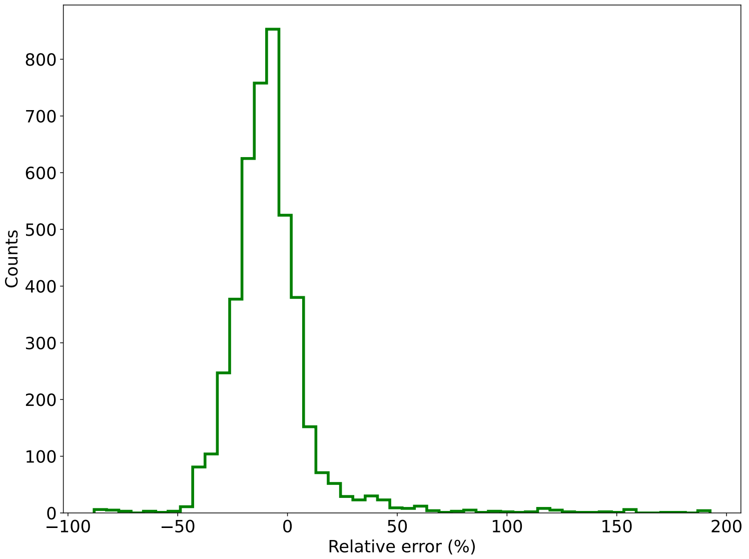

Figure 2: **Relative error between Kummu et al. GDPpc grid calculation and OECD information.** Using OECD data, we check the quality of the calculation of the metropolitan areas' GDPs through the grids. In particular, we can make this check for around 340 MAs and considering 13 years, with a total of approximately 4500 checks.

between the two datasets. To quantify, we can say that, taking the absolute value, only 2% of the checks have a relative error of GDPpc greater than 50%; only 15% have a relative error greater than 25%. More than half (55% of the checks) have a relative error of less than 15%. However, there is not a perfect match both because of the limitations given by Kummu et al.'s methodology and because of how the boundaries of the metropolitan area are chosen. The boundaries, in fact, can change depending on the methodology used for the identification of the urban area and on the year in which they were built.
Finally, we show an example of our GDPpc calculation using the grid constructed in the work of Kummu et al. [2]. In Fig. 3 we show the intersection of the grid with the MA of Rome as an example. To calculate GDPpc we average all the points (the red dots referring to the figure) within the MA and, because we are working in 5-year windows, we also average over time in each window.

3 MA data cleaning and statistical data visualization.

In the main text we described how to obtain the final matrices composed of 2169 MAs and 650 technology codes. However, to make the data cleaner, we decided to cut out from the analysis those MAs that are not very active in technology production. We filter from our dataset the metropolitan areas which, computing their total production over the years of our dataset, are relevant producers

[1] `https://stats.oecd.org/Index.aspx?DataSetCode=CITIES`

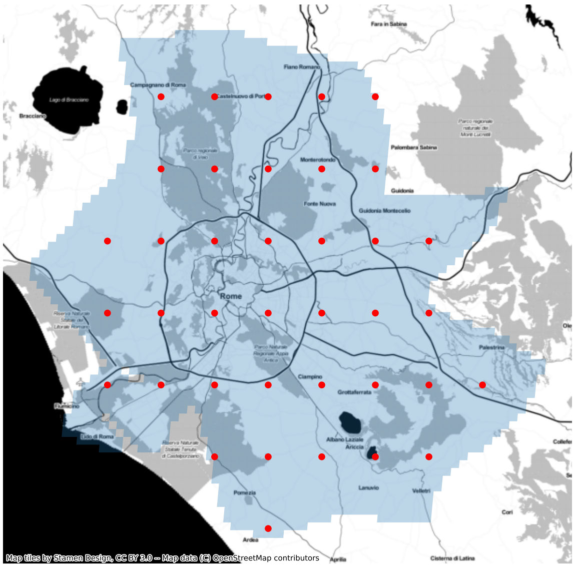

Figure 3: **Example of GDPpc grid in the MA of Rome.** The light blue area is the MA of Rome and the red dots are the part of the grid that intersects the MA.

of less than one product per year, on average, i.e. their total RCA-validated technologies are less than the number of years.

The remaining metropolitan areas are 1211 and are distributed as shown in Fig. 4 and 5. We show in these Figures the geographical distribution of MAs available to us around the world 5 and the distribution of the number of MAs for each country in the database 4. In both figures, with the red colour, we indicate the MAs in the database that are cut off (i.e. all a that have $\overline{RCA}_a < 1$), in blue those that remain (i.e. all a that have $\overline{RCA}_a \geq 1$). We are interested in capturing relatedness among technologies, and to do this we remove from the analysis the MAs that on average make very few technologies. Most of these are from emerging countries, and it will be interesting to study in future work how we can characterise their technological growth. However, in the present work, these MAs are not able to help us capture relatedness between technology codes.

4 Bipartite Configuration Model (BiCM)

Here we give a technical description of the Bipartite Configuration Model (BiCM) [3, 4] which we compute by using the *NEMtropy* Python package (`github.com/nicoloval/NEMtropy`) [5]. The BiCM belongs to the family of Exponential Random Graphs, adapted to the case of bipartite networks. These models arise from the maximisation of the Shannon entropy of an ensemble of networks, in our case undirected binary bipartite networks $\mathbf{M}$:

$$S = -\sum_{\mathbf{M}\in\Omega} P(\mathbf{M}) \ln P(\mathbf{M}),$$

considering a set of constraints $\mathbf{C}(M)$. $P(\mathbf{M})$ is the probability of a specific bipartite network $\mathbf{M}$. The probability distribution maximising the entropy is the exponential distribution:

$$P(\mathbf{M}|\vec{\lambda}) = \frac{e^{-H(\mathbf{M},\vec{\lambda})}}{Z(\vec{\lambda})}, \tag{1}$$

where $H(\mathbf{M},\vec{\lambda}) = \vec{\lambda} \cdot \mathbf{C}(M)$ is the Hamiltonian imposing the Lagrangian multipliers.

Two sets of constraints are imposed in the BiCM, one for each layer. Specifically, the node degrees

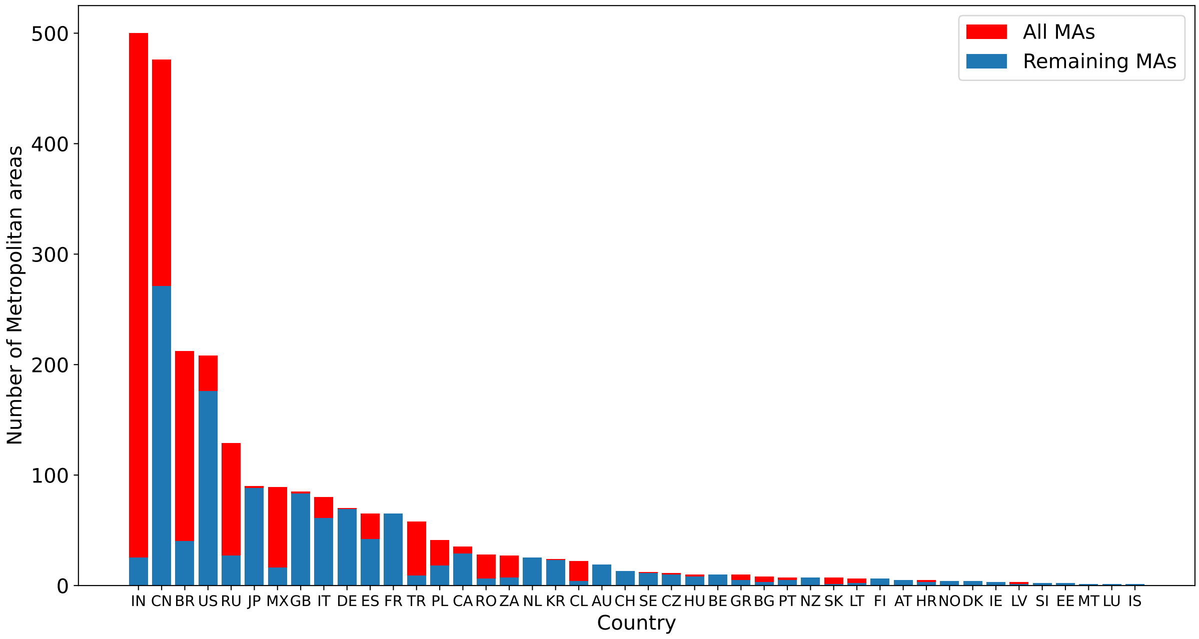

Figure 4: **Distribution of the number of MAs for each country in the database.** In red the MAs in the database that are cut off, and in blue those that remain. We use as a cut-off criterion the fact that if the sum of the RCA-validated technologies across all years of an MA is on average greater than 1 then the MA remains. We want to emphasise that we are interested in capturing relatedness among technologies, and to do this we remove from the analysis the MAs that on average make few technologies.

are fixed, namely ubiquity $\vec{u}(\mathbf{M})$ for each technology code and diversification $\vec{d}(\mathbf{M})$ for MAs, in our case. The mean values of the node degrees must be tuned to match these quantities. Then we obtain the Hamiltonian H:

$$H(\mathbf{M},\vec{\lambda}) = \vec{\alpha}\cdot\vec{d}(\mathbf{M}) + \vec{\beta}\cdot\vec{u}(\mathbf{M}).$$

Imposing the previous constraints together with the normalisation condition $\sum_{\mathbf{M}\in\Omega} P(\mathbf{M}) = 1$, we can write Eq. 1 as:

$$P(\mathbf{M}|\vec{\lambda}) = \frac{e^{-\vec{\alpha}\cdot\vec{d}(\mathbf{M})-\vec{\beta}\cdot\vec{u}(\mathbf{M})}}{\sum_{\mathbf{M}} e^{-\vec{\alpha}\cdot\vec{d}(\mathbf{M})-\vec{\beta}\cdot\vec{u}(\mathbf{M})}}.$$

Since constraints have been imposed on the mean values of the node degrees, the previous equation can be decomposed into the product of the probability distributions of a single link:

$$P(\mathbf{M}|\vec{\lambda}) = \prod_a \prod_t p_{at}^{M_{at}}\,(1-p_{at})^{1-M_{at}}$$

where $p_{at} = \frac{x_a y_t}{1+x_a y_t}$ is the probability of the link between the MA a and the technological code t, $x_a = e^{-\alpha_a}$ and $y_t = e^{-\beta_t}$. To estimate the unknown parameters we have to maximise the log-likelihood $\mathcal{L}(\vec{x},\vec{y}) = \ln P(\mathbf{M}|\vec{x},\vec{y})$, i.e. solving the system:

$$\vec{\Delta}\mathcal{L}(\vec{x},\vec{y}) = 0 \longrightarrow \begin{cases} d_a(\mathbf{M}) = \sum_t \frac{x_a y_t}{1+x_a y_t} \;\forall\; a \\ u_t(\mathbf{M}) = \sum_a \frac{x_a y_t}{1+x_a y_t} \;\forall\; t \end{cases}$$

with $d_a(\mathbf{M}) = d_a^*$ and $u_t(\mathbf{M}) = u_t^*$ representing the observed quantities.

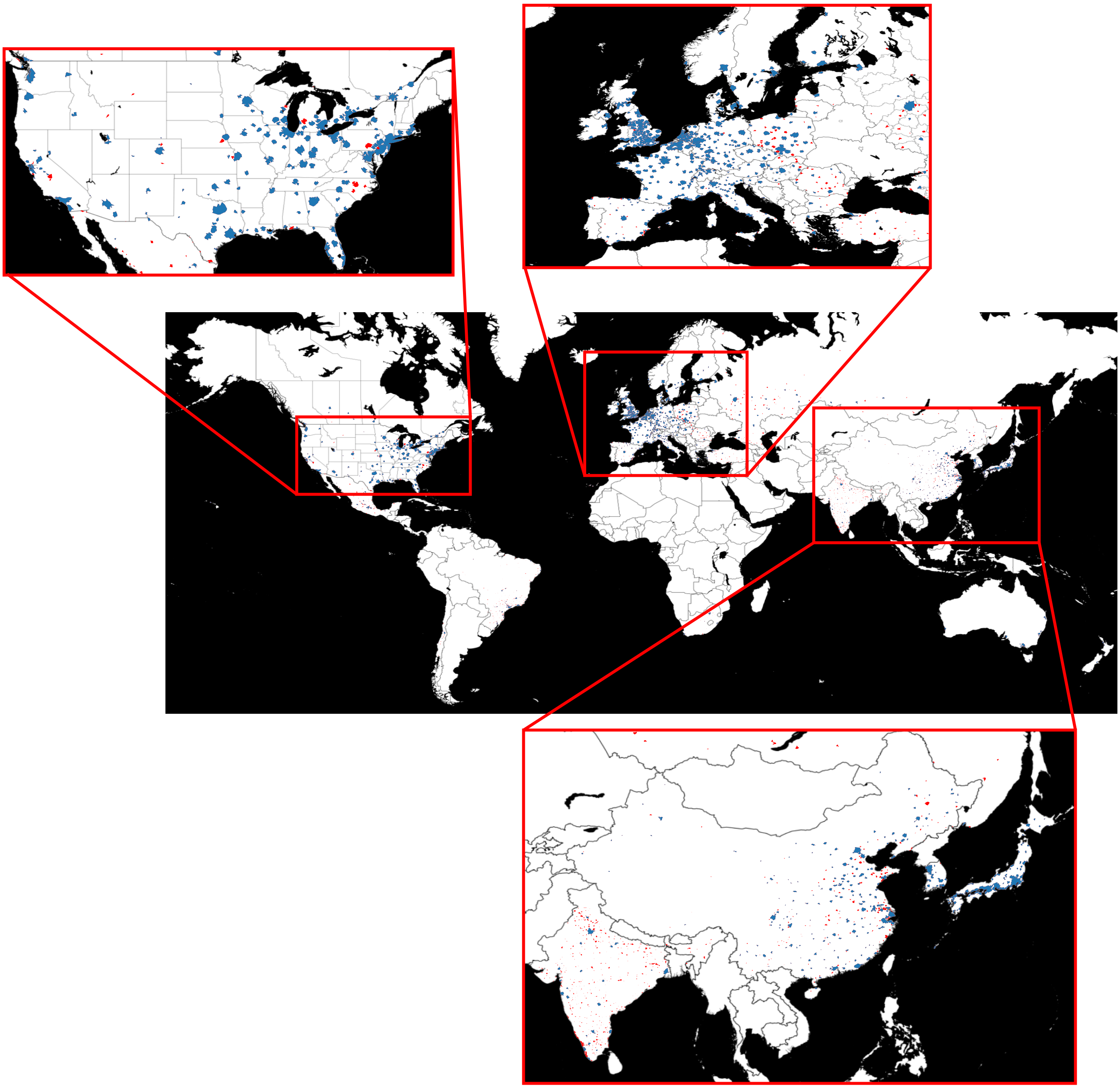

Figure 5: **Geographical distribution of MAs available to us around the world.** With red color we indicate the MAs in the database that are cut off, and in blue those that remain. We use as a cut-off criterion the fact that if the average total RCA across all years of an MA is greater than 1 then the MA remains.

After we obtain the link probabilities of the model, we use them to compute how unexpected is the number of common neighbors of two nodes of the same layer. Given that, by construction, the links of the model are independent random variables, the probability of sharing technology for two MAs is $P(V^t_{aa'} = 1) = p_{at}p_{a't}$, and the total number of technologies they share will be $V_{aa'} = \sum_t m_{at}m_{a't}$. Thus, we can compute a p-value for the number of common neighbors observed for two nodes of the same layer, which reads:

$$p\text{-value}_{aa'} = P(V_{aa'} > V^*_{aa'}) \tag{2}$$

where $V^*_{aa'}$ is the number of common neighbours between nodes a and a' in the observed network. Note that the random variable $V_{aa'}$ is a Poisson-Binomial, i.e. a sum of independent Bernoulli random variables of different parameters, which is hard to evaluate when the number of different Bernoulli is large, we actually approximate this by substituting a Poisson variable with the same mean, as it has been done in previous works.

After applying this procedure to each pair of nodes, we obtain as output a p-value matrix of the same size as the adjacency matrix $\mathbf{M}$ of the starting bipartite network. As a final step, we have to decide which of these p-values are significant and which are not. To assess the link significance, we use the False Discovery Rate test [6]: let us assume that we have N hypotheses, each characterised by its p-value. The FDR first sorts these N p-values as p-value$_1$,...,p-value$_N$, and then identifies the largest integer I such that:

$$p\text{-value}_I \leq \frac{I\alpha}{N} \tag{3}$$

where α is the arbitrarily defined single-test significance level. Note that in this case, α will be the statistical significance of the whole validated network, while for the single links, their significance will be much lower. Finally, all hypotheses with p-value lower or equal than p-value$_I$ will be rejected, i.e. the link will be validated in the projected network. In our case, for instance, in the case of the projection on the technologies' layer, the number of hypotheses is the number of possible links in the projection $\binom{N_t}{2}$ and Eq. 3 becomes:

$$p\text{-value}_I \leq \frac{I\alpha}{\binom{N_t}{2}}. \tag{4}$$

Ordering the coefficients $\binom{N_t}{2}p$-value$(V_{tt'})$ and retaining only the links between pairs of nodes t, t' such that p-value$(V^*_{tt'}) \leq p$-value$_I$ yields our projection.

Let us remark that the projection obtained via the procedure just described only keeps links that are highly significant with respect to the degree of the nodes, unveiling hidden strong similarities.

5 Networks projections

The resulting technology network was obtained by setting the parameter for the statistical validation of the projected networks of Eq. 4, to $\alpha = 0.01$ for the technologies projection. In contrast, the projected networks of MAs were obtained by setting the threshold $\alpha = 0.1$. α is a parameter setting the statistical relevance for multiple hypotheses testing, representing the statistical significance of the whole network, while the statistical significance of the single validated links is much lower (see [4] for details). The difference in the values of α for the two networks is due also to the fact that the two layers are very disproportional in their size, and setting the threshold too low in the case of MAs yields no validated links due to a high denominator in Eq.4.

After obtaining the projected networks for each time window of five years, we sum them as described in the Methods section in the main text. The two networks have a density of 0.032 and 0.012 for technologies and MAs, respectively. The mean density of the starting bipartite ones in years is 0.124. Increasing the resolution parameter in the modularity optimisation [7], we can identify three technological macro areas. These three areas correspond respectively to the light blue, pink, and olive green nodes in Fig. 6 **(a)**: The three regions contain different kinds of technologies:

- Car technologies: this region, coloured in olive green, contains technologies closely related to cars;
- Highly sophisticated technologies: this macro area, depicted in light blue, contains clusters such as electricity and communications, nuclear, and household items, all technology sectors that we can classify as highly sophisticated technology sectors;
- Manufacturing technologies: in this area, represented in pink, we can find clusters related to the textile, agri-food, plastic, and paper industries, thus containing manufacturing technology sectors.

Finally, in Fig. 6, we colour the technologies to show the average RCA values of New York (**b**) and Shanghai (**c**) in the database years. Red implies a higher RCA value, and we can note how Shanghai has focused more on manufacturing technologies while New York is strong in electricity

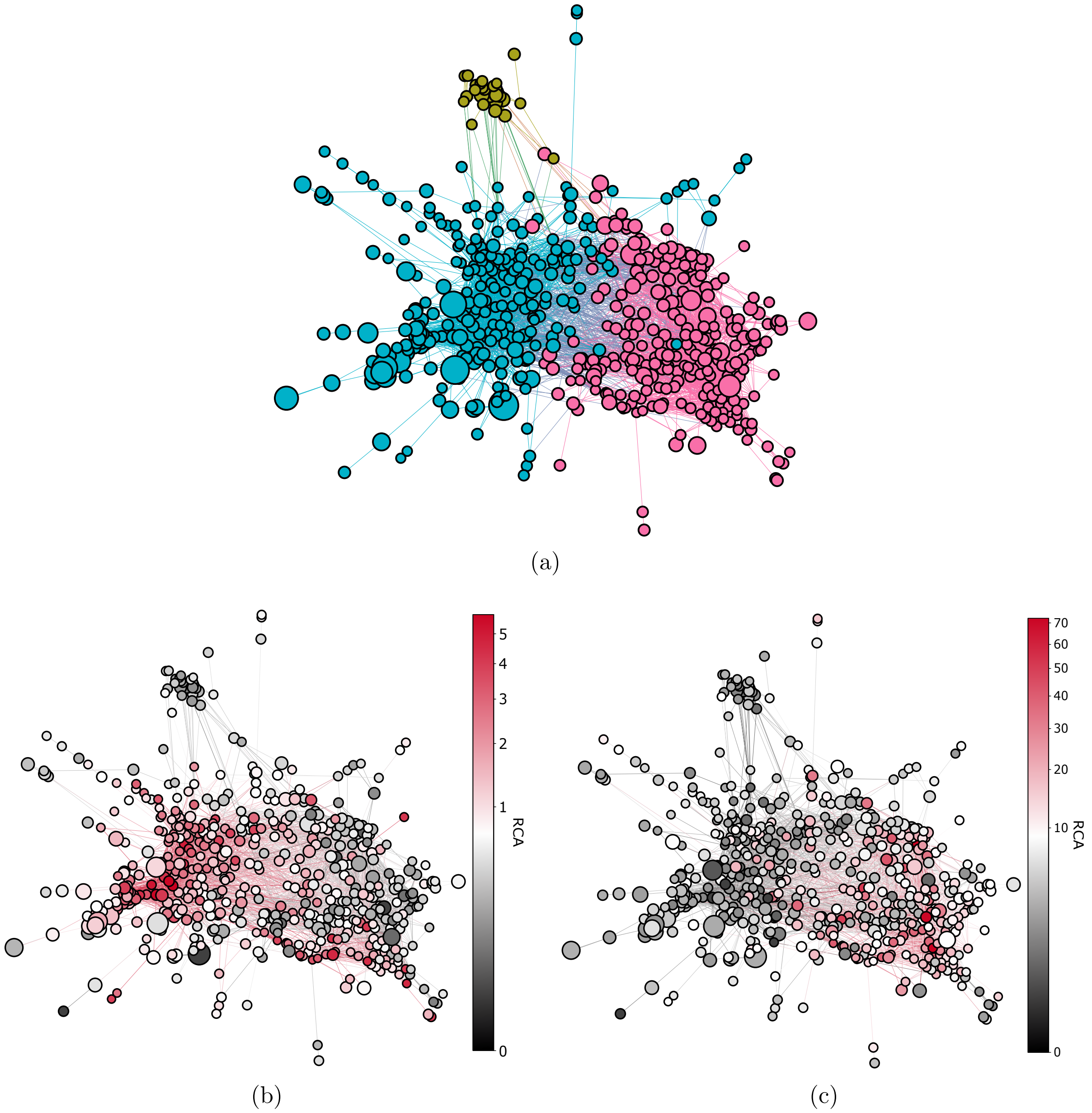

Figure 6: **Projected network of technologies.** Each node in these figures is a technology code. The size of the nodes is proportional to the complexity of the technology. **(a)**: clusters found by the community detection algorithm in the technology network with higher resolution, i.e., considering larger communities. In this case, we could identify three regions. Olive green: this region contains technologies closely related to cars; light blue: this macro area contains clusters of technology sectors that we can classify as highly sophisticated technology sectors; pink: clusters related to manufacturing technology sectors. **(b)** and **(c)**: Projection on the technology network of average RCA values in the database years of New York (**(b)**) and Shanghai (**(c)**). The colour scale shows the RCA value of the metropolitan area in a particular technology: more red nodes (technologies) indicate a high RCA value of the MA for those specific technologies. We note how Shanghai has focused more on manufacturing technologies (pink region of **(a)**), while New York is strong in electricity and communications technologies (light blue region of **(a)**).

and communications technologies.
Regarding the MA projected network, $\alpha = 0.1$ was used as the significance threshold parameter for the projection of each window; the depicted communities were found via the Louvain algorithm, and the partition features a modularity of 0.68. We apply a different significance level to the 2 layers because in the MAs network if we use a minor than 0.1 value we can't obtain nodes or links. This is a limitation of the very strict FDR threshold, but even if the 0.1 threshold is a bit higher than usual,

the significance of the single links is still very high since their p-value is lower than $0.1\frac{L}{\binom{N}{2}}$ where L, the number of links of the projection, is way smaller than the denominator, being the number of nodes in the layer. The filter becomes too strong especially when the two layers are of very different sizes.

6 Metropolitan areas community class table

In this section, we show the table in which all the MAs related to the network in Figure 3 of the main paper are present. For each community, we write the country to which the MA belongs.

Country	Community
	China Textile
CN	Changzhi
	Changzhou
	Jieyang
	Guangzhou
	Heyuan
	Huaibei
	Tianjia'an
	Deqing
	Yangzhou
	Taizhou
	Jiaxing
	Jinhua
	Jining
	Quanzhou
	Suzhou
	Nantong
	Ningbo
	Tai'an
	Yancheng
	Shuangyashan
	Xuzhou
	Jinan
	Zhenjiang
	Zhongshan
	Yichang
	Wencheng
	China high Fitness
CH	Montreux
CN	Anshan
	Anshun
	Baiyin
	Baoji
	Baotou
	Beijing
	Binzhou
	Changchun
	Taoyuan
	Changsha
	Chengdu
	Dalian
	Ranghulu
	Deyang
	Dezhou
	Dongying
	Fushun
	Fuxin
	Fuzhou
	Guilin
	Guiyang
	Haikou
	Handan
	Hangzhou
	Hefei
	Hohhot
	Xinxiang
	Huludao
	Jiaozuo
	Qingdao
	Jinchang
	Jinzhou
	Kunming
	Laiwu
	Lanzhou
	Leshan
	Lianyungang
	Liaocheng
	Linyi
	Luoyang
	Ma'anshan
	Ürümqi
	Nanchang
	Nanjing
	Nanning
	Tianjin
	Panzhihua
	Huai'an
	Qinhuangdao
	Qujing
	Shanghai
	Shenyang
	Shijiazhuang
	Gongzhuling
	Taiyuan
	Tangshan
	Dashiqiao
	Wuhan
	Xi'an
	Yinchuan
	Zhengzhou
	Qiqihar
	Zunyi
	Shizuishan
	Sanmenxia
	Nanyang
	Yantai
	Zibo
	Yuxi
	Xiamen
	Xiangtan
	Zigong
	Tongling
IN	Jamshedpur
JP	Kamisu
KR	Pohang-si
	China low Fitness
CN	Aksu
	Altay
	Ankang
	Baise
	Baishan
	Baoshan
	Bayannur
	Beihai
	Benxi
	Bozhou
	Changji/Sanji
	Chaoyang
	Chengde
	Chifeng
	Lucheng
	Chuzhou
	Dali
	Dazhou
	Huangshi
	Duyun
	Enshi
	Ganzhou
	Gongnong
	Guigang
	Guyuan
	Hanzhong
	Hebi
	Jīnchéngjiāng
	Hegang
	Hengshui
	Hengyang
	Heze
	Hezhou
	Huaihua
	Hulunbuir
	Jiamusi
	Jiayuguan
	Jilin
	Jingdezhen
	Jinghong
	Jinzhong
	Jishou
	Jiuquan
	Kaifeng
	Kaili
	Kashgar
	Hotan
	Tianshan
	Lhasa
	Liaoyang
	Linxia
	Liuzhou
	Longnan
	Louxing
	Meizhou
	Mengzi
	Nanchong
	Ordos
	Panjin
	Songyuan
	Qingyang
	Qinzhou
	Shangluo
	Shangqiu
	Shaoyang
	Suihua
	Suining
	Turpan
	Weinan
	Wenshan
	Wuwei
	Wuzhong
	Xianning
	Xichang
	Xingyi
	Xining
	Ya'an
	Yanji
	Yibin
	Yingtan
	Yongzhou
	Yuncheng
	Zhangjiajie
	Zhangye
	Xilinhot
	Sanming
	Meishan
	Yining/Qulja
	Ulanqab
	Xinzhou
	Zhongwei
	Mudanjiang
	Zhanjiang
	Zhangzhou
	Neijiang
	Zhaotong
	Blagoveshchensk
	Shiyan
	Shaoguan
	Shangrao
	Shuozhou
	Xinyu
	Panshi
	Yulin
	Ziyang
	Pingdingshan
	Rizhao
	Zhumadian
	Zhoukou
	Xuchang
	Emerging economies
AU	Hobart
BR	Recife
	Campinas
	Fortaleza
	Florianópolis
	SãoCarlos
	Ribeirao Preto
	Ceilândia
	João Pessoa
	Londrina
	Goiana
	Maringá
	Montes Claros
	Pelotas
	Viçosa
	Uberlândia
	Vitória da Conquista
CA	Sarnia
CH	Basel
CL	Concepción
CN	Sanya
	Pingliang
CZ	Hradec Králové
DE	Darmstadt
	Frankfurt am Main
	Mannheim
ES	Alacant
	Cádiz
	Córdoba
	Vitoria-Gasteiz
FR	Dunkirk
GR	Heraklion
	Patras
HU	Debrecen
	Pécs
	Szeged
IN	Lucknow
	Surat
	Madurai
	Thiruvananthapuram
	Mysuru
	Vadodara
IS	Reykjavik
IT	Palermo
	Brindisi
	Sassari
JP	Shunan
	Ube
MX	Cadereyta Jiménez
	Cuernavaca
	Guanajuato
	Hermosillo
	Saltillo
NL	Arnhem
	Maastricht
	Heerlen
NZ	Dunedin
PL	Lublin
PT	Braga
	Coimbra
RU	Saratov
US	Saginaw
	Davenport
ZA	Bloemfontein
	High Tech
AU	Melbourne
	Brisbane
CA	Toronto
	Detroit
	Ottawa
	London
ES	Girona
	Granada
FR	Marseille
	Nice
	Montpellier
GB	Bristol
	London
	Cambridge
	Middlesbrough
	Ipswich
	Plymouth
IN	Bengaluru
MX	Mexico City
NL	Eindhoven
US	San Jose
	Washington D.C.
	Baltimore
	San Antonio
	Atlanta
	Austin
	New York
	Albuquerque
	Miami
	Louisville
	Philadelphia
	Oklahoma City
	Nampa
	Charleston
	Virginia Beach
	Dallas
	Ogden
	Omaha
	Lexington
	Tallahassee
	Tampa
	Honolulu
	Los Angeles
	Phoenix
	Monterey
	Tucson
	Kansas City
	Tijuana
	Sacramento
	Palm Bay
	Seattle
	Orlando
	Bradenton
	Chicago
ZA	Cape Town
	Korea & Japan
CA	Guelph
CN	Anyang
	Jinan
EE	Tallinn
ES	Seville
	Murcia
	Palma de Mallorca
	Almeria
FR	La Rochelle
	Aix-en-Provence
	Avignon
	Clermont-Ferrand
JP	Tomakomai
	Kagoshima
	Aomori
	Asahikawa
	Naha
	Fukuoka
	Sapporo
	Shizuoka
	Hachinohe
	Hakodate
	Kōchi
	Yonago
	Izumo
	Kitami
	Kushiro
	Obihiro
	Kan'onji
	Morioka
	Hirosaki
	Miyakonojo
	Niigata
	Ōmura
	Izumimachikurosuno
	Ōita
KR	Seoul
	Cheonan-si
	Busan
	Cheongju-si
	Chuncheon-si
	Chungju-si
	Daegu
	Gwangju
	Iksan-si
	Jeonju
	Mokpo-si
	Wonju-si
	Gunsan-si
	Sora-myeon
NL	Breda
NZ	Hamilton
	Western
AU	Gold Coast
CA	Abbotsford
	Barrie
	Red Deer
	Winnipeg
	Kingston
ES	A Coruña
	Logroño
	Zaragoza
	Gijón
	Lleida
FI	Jyväskylä
	Kuopio
FR	Ajaccio
	Perpignan
	Metz
	Angers
	Troyes
	Le Mans
	Lorient
	Toulon
	Béziers
	Cherbourg
	Saint-Brieuc
GB	Brighton
	Ashford
	Southend-on-Sea
	Blackburn
	Blackpool
	Bournemouth
	Bridgend
	Norwich
	Cardiff
	Carlisle
	Swansea
	Crewe
	Preston
	Colchester
	Corby
	Exeter
	Portsmouth
	Stoke-on-Trent
	Eastbourne
	Newcastle upon Tyne
	Great Yarmouth
	St Leonards
	Peterborough
	Lancaster
	Lincoln
	Luton
	Grimsby
	Scunthorpe
	Fazeley
	Redditch
	Taunton

	Torquay
	Royal Tunbridge Wells
	Chelmsford
	Dunfermline
	Chester
	Glasgow
	Liverpool
IT	Bolzano - Bozen
NL	Hoorn
NZ	Auckland
TR	Kapaklı
US	Anchorage
	Killeen
	Waterloo
	Bismarck
	Chico
	Shreveport
	Lake Charles
	Cape Coral
	Naples
	Fort Smith
	Winston-Salem
	Dubuque
	Palm Coast
	Flint
	Port Arthur
	Spring Hill
	Kankakee
	La Crosse
	Lubbock
	Wilkes-Barre
	Madera
	Port St. Lucie
	Boston
	Lake Havasu City
	Corpus Christi
	Navarre
	Brockton
	Amarillo
	Tracy
	Topeka
	Modesto
	Abilene
	Visalia
	Yakima
	Yuma
	Deltona
	Murfreesboro
	Jacksonville
	Nuevo Laredo
	Saint Cloud
	Wichita Falls
	Savannah
	Salt Lake City
ZA	Durban
	Western Cars
DE	Stuttgart
	Ingolstadt
FR	Montbéliard
GB	Birmingham
	Coventry
	Warwick
IT	Turin
	Reggio nell'Emilia
JP	Nagoya
	Hamamatsu
US	Detroit
	Peoria
	Janesville
	Ann Arbor

7 Coherence robustness test

In this section, we present the Coherence robustness test presented in the main text. This consists of showing the diversification distributions of MAs and calculating the technology network without Chinese MAs.
In figure 7, we plot the three distributions. It might happen that Chinese MAs produce few tech-

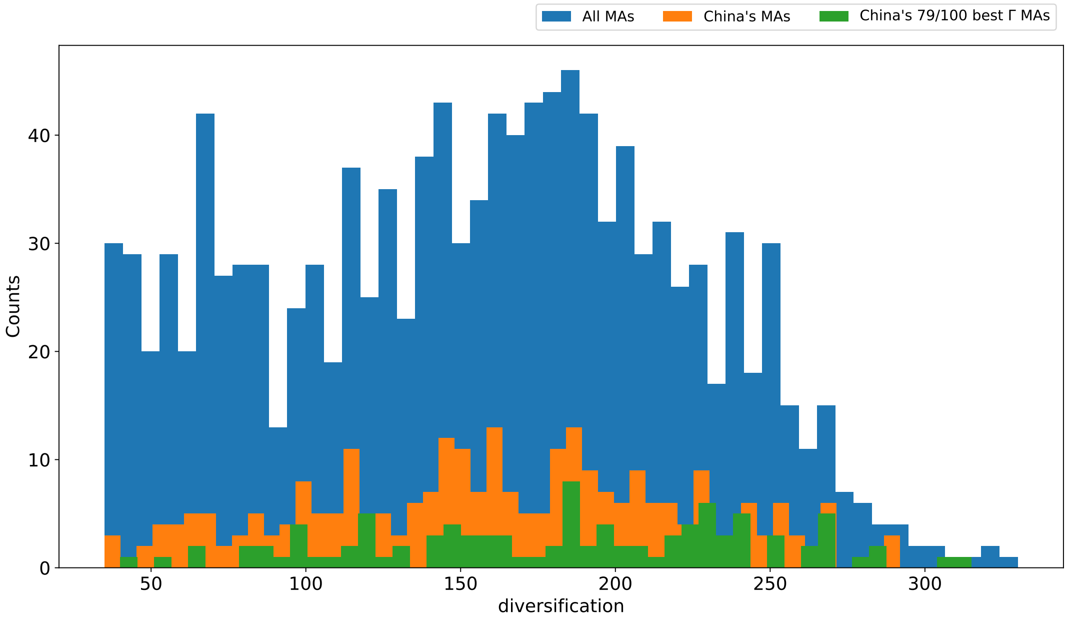

Figure 7: **Diversification distribution.** We plot respectively the distribution of all the MAs, Chinese ones, and the 79 higher Coherence ones, to show that the high Coherence is not the cause of the low diversification.

nologies which are closely related; on the other hand, from the trend (green distribution in Figure 7) we see that the 79 Chinese MAs that show a high consistency value are distributed across the diversification spectrum.
In the "technology network without Chinese MAs test", we recalculate the network of technologies in the same way that the one in the main text was calculated, except that we do so by removing all Chinese MAs. In Figure 8 we show a representation of the network of technologies obtained without

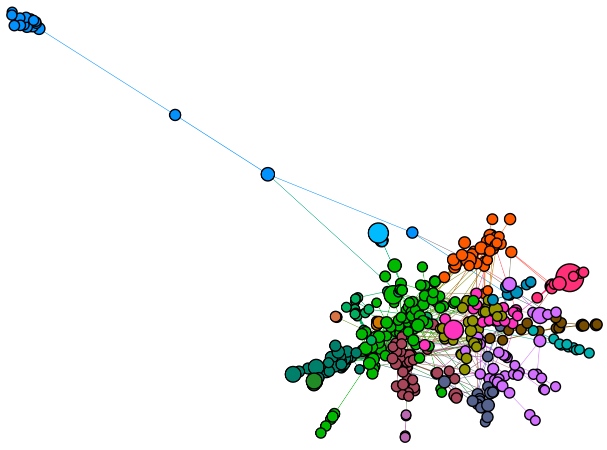

Figure 8: **Technology code network obtained without Chinese MAs.** The size of the nodes is proportional to the complexity of the technology.

the Chinese MAs. Starting from this, finally, we can compute the Coherence of the diversification of MAs by Equations 6 and 7 in the main text.
Finally, in Figure 9 we show the Coherence Γ VS Fitness F plane. Values are the respective average over the decade 1995-2005 and the color scale is the percentage change of GDPpc over these years. We represent Chinese MAs with star markers. Considering the ranking of Γ for MAs, it appears

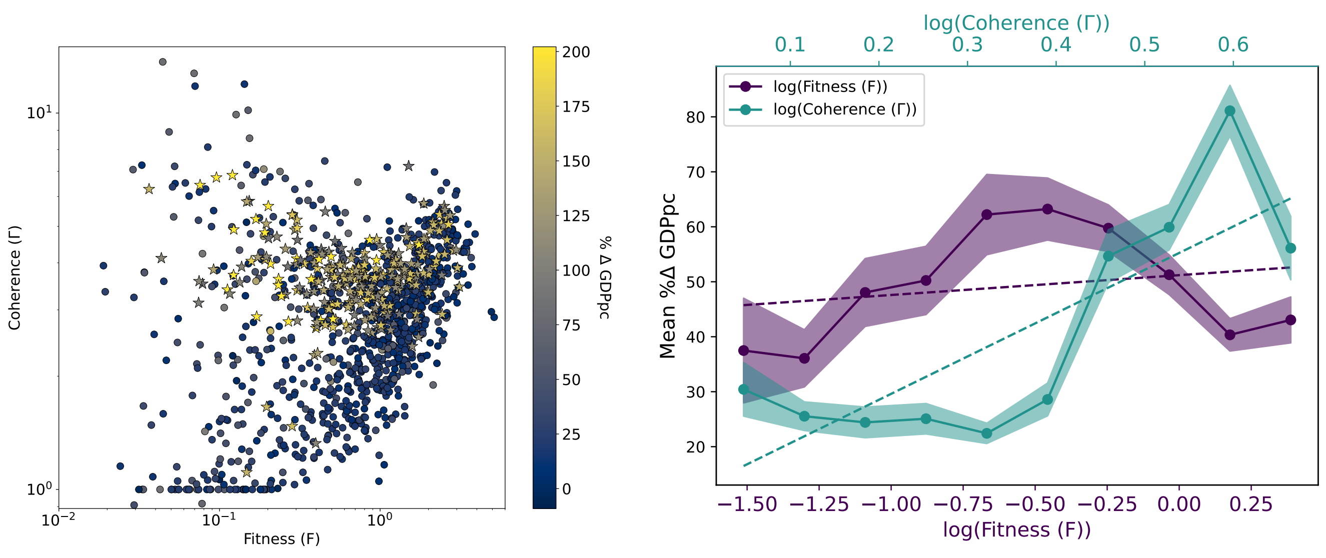

(a) (b)

Figure 9: **Fitness VS Coherence to evaluate GDPpc growth.** **(a)** Coherence Γ VS Fitness F plane. Coherence coefficients (i.e. product similarities) are computed after removing Chinese MAs from the system. Values are the respective average over the decade 1995-2005 and the color scale is the percentage change of GDPpc over these years. With star markers, we are indicating the Chinese MAs. Considering the ranking of Γ for MAs, it appears that of the top 100, 24 are Chinese ones. **(b)** Average GDPpc versus Fitness and Coherence. Again, Coherence coefficients are computed after removing Chinese MAs from the system. To highlight that Coherence is better able to discriminate change in GDPpc, we divide both Fitness and Coherence into ten bins and calculate the mean GDPpc variation of all the points in each of the ten bins. We show how the Fitness curve is roughly constant while the Coherence one has an increasing trend. This means that, on average, looking at the Fitness, we will find approximately the same value of % Δ GDPpc while looking at the Coherence, the higher this one is, the higher the variation of GDPpc. We show also the lines of the best fit of both curves to highlight the difference between the two trends.

that of the top 100, 24 are Chinese ones. To evidence that Coherence is better able to discriminate change in GDPpc, we divide both Fitness and Coherence into ten bins and calculate the mean GDPpc variation of all the points in each of the ten bins in Fig. 9**b**. We show how the Fitness curve is roughly constant while the Coherence one has an increasing trend. This means that, on average, looking at the Fitness, we will find approximately the same value of % Δ GDPpc while looking at the Coherence, the higher this one is, the higher the variation of GDPpc. We show also the lines of the best fit of both curves to highlight the difference between the two trends.

8 Coherence of clusters

In Figure 10, we show the average Coherence for each of the clusters we found, obtained by averaging all Coherence values of the MAs in the respective clusters.

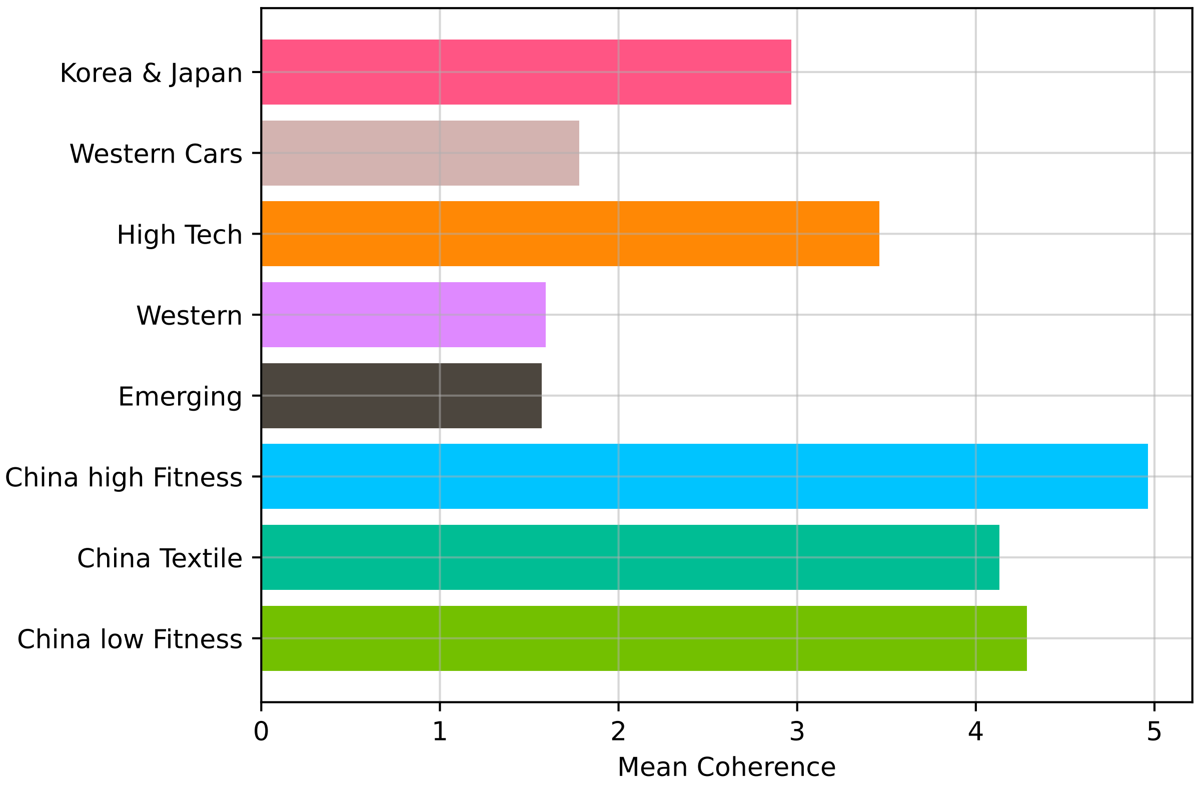

Figure 10: **The mean Coherence of each cluster of metropolitan areas.** The clusters containing Chinese cities have the highest average Coherence, and high Fitness clusters tend to have higher values of Coherence as well.

Confirming our findings, the Chinese clusters still show the highest levels of Coherence. High Fitness clusters seem to have higher values of Coherence: the high Fitness clusters such as high tech or the Japanese/Korean one show on average a higher Coherence than the other non-Chinese clusters.

References

1. Gaétan De Rassenfosse, Jan Kozak, and Florian Seliger. Geocoding of worldwide patent data. *Scientific data*, 6(1):1–15, 2019.

2. Matti Kummu, Maija Taka, and Joseph HA Guillaume. Gridded global datasets for gross domestic product and human development index over 1990–2015. *Scientific data*, 5(1):1–15, 2018.

3. Fabio Saracco, Riccardo Di Clemente, Andrea Gabrielli, and Tiziano Squartini. Randomizing bipartite networks: the case of the world trade web. *Scientific Reports*, 5(1):1–18, 2015.

4. Fabio Saracco, Mika J Straka, Riccardo Di Clemente, Andrea Gabrielli, Guido Caldarelli, and Tiziano Squartini. Inferring monopartite projections of bipartite networks: an entropy-based approach. *New Journal of Physics*, 19(5):053022, 2017.

5. Nicolò Vallarano, Matteo Bruno, Emiliano Marchese, Giuseppe Trapani, Fabio Saracco, Giulio Cimini, Mario Zanon, and Tiziano Squartini. Fast and scalable likelihood maximization for exponential random graph models with local constraints. *Scientific Reports*, 11(1):1–33, 2021.

6. Yoav Benjamini and Yosef Hochberg. Controlling the false discovery rate: a practical and powerful approach to multiple testing. *Journal of the Royal statistical society: series B (Methodological)*, 57(1):289–300, 1995.

7. Renaud Lambiotte, J-C Delvenne, and Mauricio Barahona. Laplacian dynamics and multiscale modular structure in networks. *arXiv:0812.1770*, 2008.
